# Upregulation of microRNA-196a and microRNA-196b cooperatively correlate with aggressive progression and unfavorable prognosis in patients with colorectal cancer

**DOI:** 10.1186/s12935-014-0128-2

**Published:** 2014-12-14

**Authors:** Jie Ge, Zihua Chen, Ruixing Li, Tailiang Lu, Guangfa Xiao

**Affiliations:** Department of General Surgery, Xiangya Hospital, Central South University, 87# Xiangya Road, Changsha, Hunan 410008 China

**Keywords:** Colorectal cancer, MicroRNA-196a, microRNA-196b, Clinicopathological characteristics, Overall survival, Disease free-survival

## Abstract

**Background:**

Both microRNA (miR)-196a and miR-196b are implicated in normal cell differentiation, proliferation, and in tumorigenesis of various cancer types. Especially, miR-196a exerts a pro-oncogenic influence in colorectal cancer (CRC) cells and miR-196b expression is upregulated in CRC tissues. The aim of this study was to evaluate the associations of miR-196a and miR-196b dysregulation with clinicopathological characteristics and prognosis in patients with CRC.

**Methods:**

Quantitative real time-PCR (qRT-PCR) was performed to detect the expression levels of miR-196a and miR-196b in 126 pairs of fresh tumor samples matched with adjacent colorectal mucosa obtained from 126 patients with CRC.

**Results:**

miR-196a and miR-196b expression levels in CRC tissues were significantly higher than those in adjacent colorectal mucosa (both P < 0.002). Interestingly, the expression levels of miR-196a in CRC tissues were positively correlated with those of miR-196b. Then, high miR-196a expression and high miR-196b expression, alone or in combination, were all statistically linked to the presence of lymph node metastasis, the poor differentiation grade, and the advanced TNM stage of CRC. Moreover, overall and disease-free survivals of CRC patients with high miR-196a expression, high miR-196b expression and miR-196a-high/miR-196b-high expression tended to be shorter than the corresponding control groups (log-rank statistic, all P < 0.001). Furthermore, multivariate analysis indicated miR-196a and/or miR-196b expression as independent prognostic indicators for CRC patients (all P < 0.05).

**Conclusions:**

Both miR-196a and miR-196b may be correlated with aggressive progression and unfavorable clinical outcome in CRC patients. Combined expression of miR-196a and miR-196b may be a promising biomarker in identifying a poor prognosis group of CRC.

## Background

Colorectal cancer (CRC) represents the third most common malignant tumor and the second leading cause of cancer-related death worldwide [1]. The incidence of CRC has been increasing in Asian countries, such as China, Korea, Japan and Singapore, due to demographic trends and adaption to westernized lifestyle in developing countries [2]. Although the 5-year overall survival rate of CRC patients has been reported to be as high as 71.3%, the survival rate in patients with recurrence is only 40% [3]. Nearly 50% of CRC patients die from distant metastases within 6 years of the initial diagnosis, and the liver is the predominant and often the only organ for distant metastasis [4]. The recurrence rate of CRC patients who received curative resection has been reported to be 27.3% [5]. Besides radical surgery, adjuvant therapies such as chemotherapy and targeted therapy have been widely used. However there has been no breakthrough in the control of CRC once it develops extra lymph node metastasis. Although several clinicopathological characteristics, such as tumor size, TNM stage, differentiation grade, lymph node metastasis and tumor invasion, have been reported to evaluate CRC patients’ prognosis, it still varies greatly among patients with the same clinicopathological characteristics [6]. Therefore, it is extremely necessary to screen novel and efficient biomarkers which can be used to predict recurrence in order to choose more appropriate treatment for each CRC patients.

microRNAs (miRNAs) belong to a broad class of small non-coding RNAs usually with 21–25 nucleotides in length and functionally regulate gene expression at the post-transcriptional level through base pairing to partially complementary sites [7]. The discovery of miRNAs may be one of the most significant advances in biological and medical sciences in the last decade. Until now, hundreds of miRNAs have been identified in plants, viruses, animals and human beings [8]. miRNAs have been demonstrated to be implicated in multiple cellular processes, including development, cell differentiation, cell proliferation, apoptosis, stem cell maintenance, and epithelial–mesenchymal transition [9]. Besides these biological functions, miRNAs have also been reported to be involved in a variety of pathological processes, such as human cancers. Accumulating studies have shown widespread alteration of miRNA expression patterns in cancer. It has also been found that in cancer global miRNA expression profiles, as well as expression of specific miRNAs, correlate with patients’ prognosis. Depending on the functions of their target genes, miRNAs can act either as oncogenes or tumor suppressors. Tumor-suppressive miRNAs usually repress growth-promoting genes, and oncogenic miRNAs often target cell growth inhibiting genes. Especially, an increasing number of studies have detected various miRNAs with aberrant expression in CRC, and these aberrant expression patterns can accurately differentiate CRC from benign colorectal mucosa, implying the potential clinical values of miRNAs in CRC [10–12].

The miR-196 family consists of miR-196a and miR-196b and mature miR-196b differs from miR-196a by one nucleotide [13]. Functionally, this family has been reported to play crucial roles in normal development and cancer pathogenesis by regulating its target genes. The expression levels of members in the miR-196 family have been found to be strictly controlled under physiologic conditions, whereas dysregulation of miR-196s has also been observed in many disease conditions [14,15]. Depending on their direct targets, the miR-196 family exerts either an oncogenic function or a tumor suppressive role in various cancers. Schimanski et al. [16] indicated that high levels of miR-196a could promote CRC cell detachment, migration, invasion and diminish chemosensitivity towards platin derivatives, and also could increase the development of lung metastases in mice after tail vein injection, implying a pro-oncogenic influence of miR-196a in CRC; Wang et al. [17] used Agilent microRNA microarrays consisting of 723 probes to screen the expression differences of miRNAs, and found that miR-196b was the one of upregulated miRNAs in CRC tissues compared with the para-cancerous controls. These previous data suggest that miR-196a and miR-196b may be involved in tumorigenesis and tumor progression of CRC. However, their clinical significance in this disease remains unclear. This study aimed to evaluate the associations of miR-196a and miR-196b dysregulation with clinicopathological characteristics and prognosis in patients with CRC.

## Results

### Upregulation of miR-196a and miR-196b in human CRC tissues

**Figure 1:**
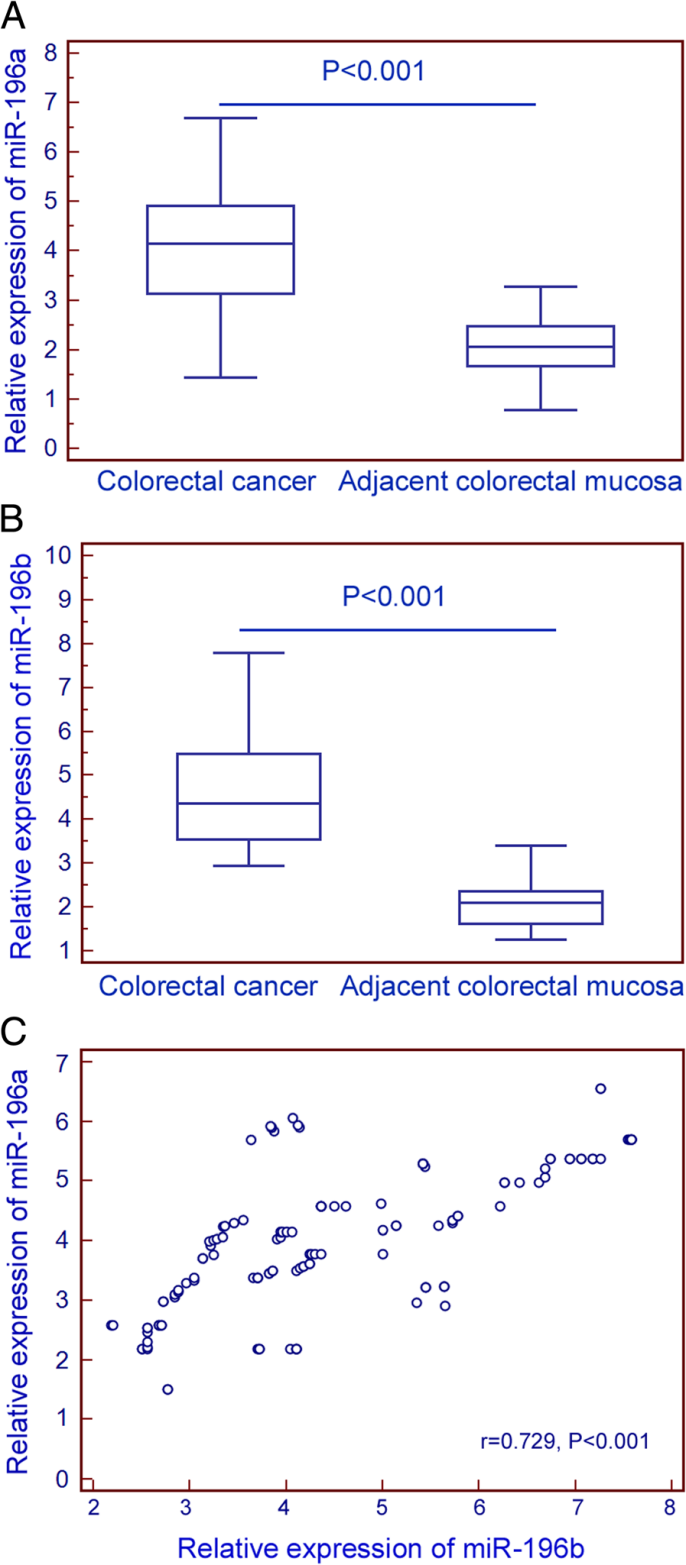
**Expression levels of miR-196a and miR-196b in colorectal cancer tissues.** Relative expression levels of miR-196a **(A)** and miR-196b **(B)** in colorectal cancer tissues and adjacent colorectal mucosa. The expression levels of miR-196a in colorectal cancer tissues were positively correlated with those of miR-196b significantly (Spearman correlation coefficient r = 0.729, P < 0.001, **C**).

miR-196a (Cancer vs. Normal: 4.21 ± 1.66 vs. 2.06 ± 1.01, P < 0.001, Figure 1A) and miR-196b (Cancer vs. Normal: 4.38 ± 1.81 vs. 2.11 ± 1.02, P < 0.001, Figure 1B) expression were found significantly upregulated in CRC compared to adjacent colorectal mucosa. Interestingly, the expression levels of miR-196a in CRC tissues were positively correlated with those of miR-196b significantly (Spearman correlation coefficient r = 0.729, P < 0.001, Figure 1C).

Then, we chose the median values of miR-196a (4.18) and miR-196b (4.33) expression as the cutoff points. CRC patients with an expression level exceeding the median values for miR-196a or miR-196b were deemed to be high expressions of miR-196a or miR-196b; all other patients were considered to be low expressions of miR-196a or miR-196b. Among 126 patients with CRC, 52 (41.27%) both highly expressed miR-196a and miR-196b (miR-196a-high/miR-196b-high), 24 (19.05%) both lowly expressed miR-196a and miR-196b (miR-196a-low/miR-196b-low), 24 (19.05%) were miR-196a-high and miR-196b-low expression (miR-196a-high/miR-196b-low), and 26 (20.63%) were miR-196a-low and miR-196b-high expression (miR-196a-low/miR-196b-high).

### Associations of miR-196a and/or miR-196b upregulation with the clinicopathological features of human CRC

The associations of miR-196a and/or miR-196b upregulation (miR-196a-high/miR-196b-high) with the clinicopathological features of CRC were also evaluated as summarized in Table 1. High miR-196a expression and high miR-196b expression were both significantly associated with the presence of lymph node metastasis (both P = 0.01, Table 1), poor differentiation grade (both P = 0.02, Table 1) and advanced TNM stage (both P = 0.006, Table 1). Of 126 CRC patients, 52 (41.27%) belonged to the miR-196a-high/miR-196b-high group in which more frequently occurred lymph node metastasis (P = 0.008, Table 1), and had poor differentiation grade (P = 0.01, Table 1) and advanced TNM stage (P < 0.001, Table 1) than those with other groups with different expression patterns for these two miRNAs. However, there was no significant associations between patients’ gender, age, site of primary tumor, tumor size or depth of tumor invasion, and the expression patterns of miR-196a and/or miR-196b.

**Table 1:** **Associations of miR-196a and/or miR-196b expression with the clinicopathological characteristics of colorectal cancer**

**Clinical features**	**Case number (%)**	**miR-196a-high**		**miR-196b-high**		**miR-196a-high/miR-196b-high**	
		**(N, %)**	**P**	**(N, %)**	**P**	**(N, %)**	**P**
**Gender**							
Male	76 (60.32)	46 (60.53)	NS	48 (63.16)	NS	31 (40.79)	NS
Female	50 (39.68)	30 (60.00)		30 (60.00)		21 (42.00)	
**Age (years)**							
<66	48 (30.10)	28 (58.33)	NS	28 (58.33)	NS	21 (43.75)	NS
≥66	78 (69.90)	48 (61.54)		50 (64.10)		31 (39.74)	
**Site of primary tumor**							
Right-sided	48 (38.10)	30 (62.50)	NS	30 (62.50)	NS	20 (41.67)	NS
Left-sided	46 (36.51)	28 (60.87)		28 (60.87)		20 (43.48)	
Rectum	32 (25.40)	18 (56.25)		20 (62.50)		12 (37.50)	
**Tumor size (cm)**							
<5	70 (55.56)	40 (57.14)	NS	42 (60.00)	NS	30 (42.86)	NS
≥5	56 (44.44)	36 (64.29)		36 (64.29)		22 (39.29)	
**Depth of tumor invasion**							
T1-T2	47 (37.30)	26 (55.32)	NS	28 (59.57)	NS	20 (42.55)	NS
T3-T4	79 (62.70)	50 (63.29)		50 (63.29)		32 (40.51)	
**Lymph node metastasis**							
Negative	68 (53.97)	24 (35.29)	0.01	26 (38.24)	0.01	12 (17.65)	0.008
Positive	58 (46.03)	52 (89.66)		52 (89.66)		40 (68.97)	
**TNM stage**							
I	13 (10.32)	0 (0)	0.006	0 (0)	0.006	0 (0)	<0.001
II	46 (36.51)	20 (43.48)		22 (47.83)		10 (18.87)	
III	50 (39.68)	39 (78.00)		39 (78.00)		25 (50.00)	
IV	17 (13.49)	17 (100.00)		17 (100.00)		17 (100.00)	
**Histological grade**							
Well/moderately	80 (63.49)	40 (50.00)	0.02	40 (50.00)	0.02	22 (27.50)	0.01
Poorly	46 (36.51)	36 (78.26)		38 (82.61)		30 (65.22)	

Note: ‘NS’ refers to the difference without statistical significance.

### Prognostic implications of miR-196a and/or miR-196b upregulation in human CRC

**Figure 2:**
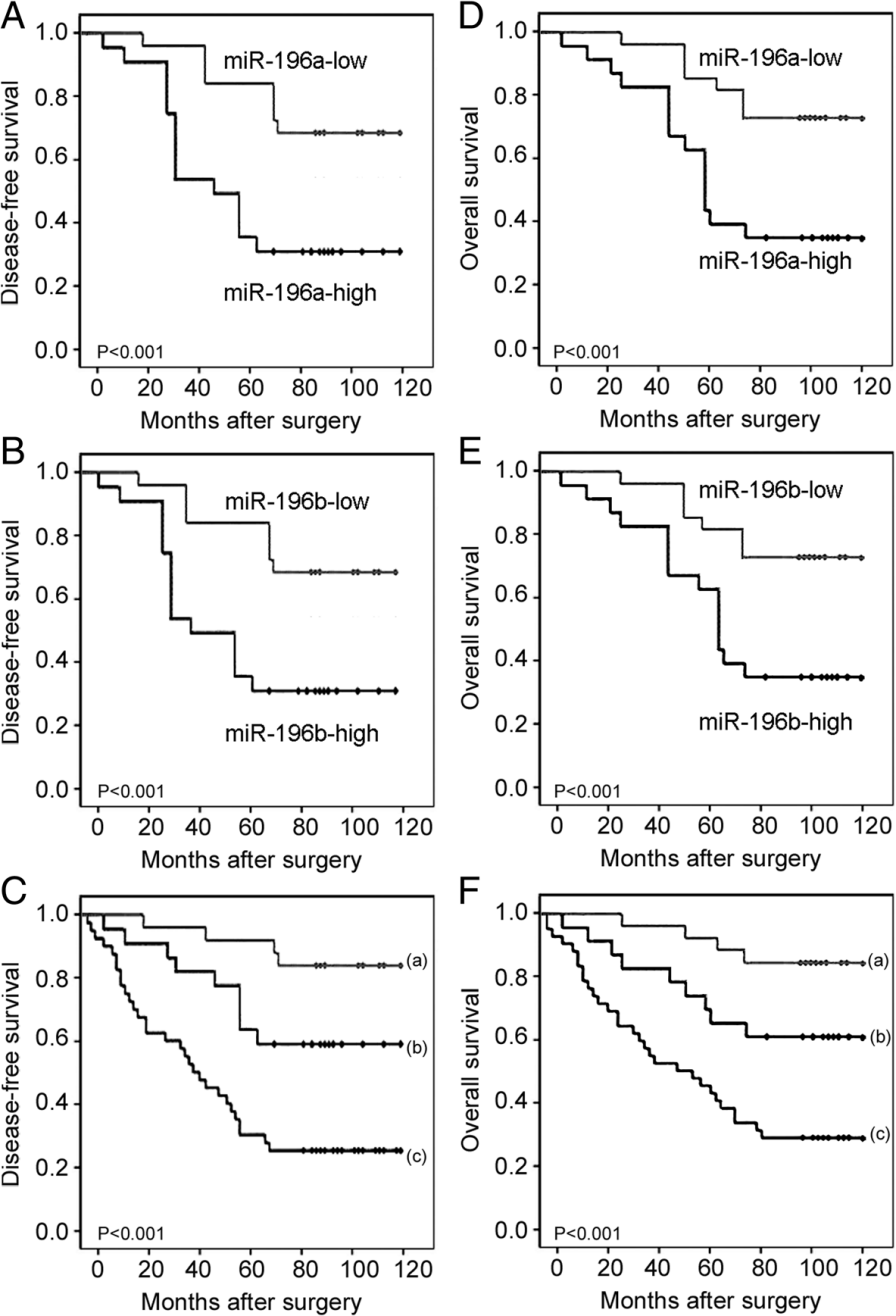
**Prognostic value of miR-196a and miR-196b in colorectal cancer.** Kaplan-Meier survival curves of the disease free survival (**A** for miR-196a expression, **B** for miR-196b expression, **C** for miR-196a/miR-196b expression) and overall survival (**D** for miR-196a expression, **E** for miR-196b expression, **F** for miR-196a/miR-196b expression) for miR-196a and/or miR-196b expression in colorectal cancer. ‘a’ refers to miR-196a-low/miR-196b-low group; ‘b’ refers to miR-196a-low/miR-196b-high & miR-196a-high/miR-196b-low groups; ‘c’ refers to miR-196a-high/miR-196b-high group.

We further investigated the potential prognostic impacts of miR-196a and/or miR-196b upregulation in CRC by the Kaplan-Meier method. Our assessment revealed that disease-free survival (DFS) and overall survival (OS) of CRC patients with high miR-196a expression, high miR-196b expression and miR-196a-high/miR-196b-high were all shorter than those of patients with low miR-196a expression, low miR-196b expression and miR-196a-high (low)/miR-196b-low (high) and miR-196a-low/miR-196b-low expression (all P < 0.001, Figure 2). Of note, CRC patients with miR-196a-high/miR-196b-high had the worst DFS and OS.

Univariate analysis showed that lymph node metastasis, TNM stage, histological grade, and miR-196a and/or miR-196b expression were factors with a significant impact on DFS and OS (Table 2). In multivariate analysis, lymph node metastasis, TNM stage, histological grade, and miR-196a and/or miR-196b expression were all identified as independent prognostic factors for both DFS and OS in patients with CRC (all P < 0.05, Table 2). We also confirmed that the combination of miR-196a and miR-196b is a better prognostic factor than miR-196a or miR-196b alone.

**Table 2:** **Prognostic value of miR-196a and/or miR-196b expression for the disease free survival and overall survival in univariate and multivariate analyses by Cox Regression**

	**Disease free survival**		**Overall survival**	
	**Hazard ratio (95% CI)**	**P**	**Hazard ratio (95% CI)**	**P**
**Univariate**				
Gender	1.005 (0.101-2.208)	NS	1.298 (0.122-2.891)	NS
Age	1.091 (0.198-2.623)	NS	1.255 (0.226-2.879)	NS
Site of primary tumor	0.698 (0.119-5.5196b)	NS	0.869 (0.126-1.881)	NS
Tumor size	0.379 (0.103-0.743)	NS	0.799 (0.136-1.606)	NS
Depth of tumor invasion	0.462 (0.102 -1.039)	NS	0.729 (0.108 -1.692)	NS
Lymph node metastasis	3.232 (1.566-6.676)	0.01	3.528 (1.6196b-7.869)	0.008
TNM stage	5.069 (1.981-12.620)	<0.001	5.869 (1.996-12.788)	<0.001
Histological grade	3.982 (1.119-8.1196b)	0.006	4.569 (1.126-10.281)	0.001
miR-196a expression	4.962 (1.663-10.612)	<0.001	4.996 (1.682-10.138)	<0.001
miR-196b expression	4.903 (1.616-10.299)	<0.001	4.969 (1.618-10.210)	<0.001
miR-196a/miR-196b	5.287 (1.970-12.396)	<0.001	5.899 (1.981-12.902)	<0.001
**Multivariate**				
Lymph node metastasis	2.162 (1.112-4.862)	0.02	2.681 (1.193-5.628)	0.01
TNM stage	4.619 (1.882-10.206)	0.001	4.689 (1.896-10.886)	0.001
Histological grade	2.682 (1.122-5.616)	0.01	2.892 (1.128-5.918)	0.01
miR-196a expression	4.668 (1.632-10.261)	0.001	4.691 (1.688-10.318)	0.001
miR-196b expression	4.532 (1.569-9.696)	0.001	4.696 (1.618-10.110)	0.001
miR-196a/miR-196b	5.106 (1.920-10.638)	<0.001	5.292 (1.961-10.692)	<0.001

## Discussion

Despite an increasing number of successful systemic therapies for cancer treatment, the clinical outcome of CRC patients with recurrence or metastasis is still poor and the long-term survival rates remain low [18]. To date, a growing list of biomolecular, genetic or cytogenetic attributes of tumors have been reported to be associated with progression and prognosis in CRC over the past two decades [19]. However, the majority of biomarkers identified so far have failed to be validated in clinical studies. Thus, new biomarkers to detect early metastasis and to predict recurrence in CRC are in urgent demand. In the current study, our data indicated that miR-196a and miR-196b expression were found to be not only significantly upregulated in CRC compared to corresponding normal colorectal mucosa, but also closely correlated with tumor progression and prognosis in patients with CRC. These findings are compatible with the hypothesis that aberrant expression of miR-196a and miR-196b may render CRC more aggressive, more prone to metastasis formation and recurrence. To the best of our knowledge, this is the first study showing the clinical value of miR-196a and miR-196b expression in a large series of patients with CRC.

The miR-196 family of miRNAs is located at three paralogous loci in the mammalian HOX clusters which encodes homoeodomain-containing transcription factors regulating differential genetic programs during embryonic development [20]. Recent studies have indicated that miR-196s are aberrantly expressed in several tumor tissue samples, and play various roles in different cancer types through targeting specific genes [21]. As a member of the miR-196 family, miR-196a expression is influenced by regulatory controls imposed on the HOX clusters [22]. It has been demonstrated that miR-196a expression is increased in several human cancers, including leukaemia, oesophageal adenocarcinoma, breast cancer, non-small cell lung cancer, pancreatic cancer, CRC and cervical cancer [16,23–26]. Functionally, it acts as an oncogenic miRNA in these malignancies. For example, miR-196a promotes pancreatic cancer progression by targeting nuclear factor kappa-B-inhibitor alpha [24]; miR-196a promotes non-small cell lung cancer cell proliferation and invasion through targeting HOXA5 [23]; miR-196a promotes cervical cancer proliferation through the regulation of FOXO1 and p27Kip1 [25]; miR-196a promotes CRC cell detachment, migration, invasion and chemosensitivity towards platin derivatives via regulating HoxA7, HoxB8, HoxC8 and HoxD8 [16]. These reports clearly reveal target specific roles of miR-196a in various human cancer types. Regarding another member of the miR-196 family, the expression pattern and functional role of miR-196b are very controversial across various cancers. miR-196b is closely associated with some types of leukemia and functions as a tumor suppressor [27]. Enforced expression of miR-196b leads to reduced cell growth, clonogenicity, migration and invasion in vitro, as well as reduced tumor angiogenesis and tumor cell proliferation in vivo via regulating the Homeobox B7-vascular endothelial growth factor axis [28]. In contrast, miR-196b expression is elevated in glioblastoma and negatively correlated with overall survival of patients with this disease [29]. Overexpression of miR-196b induces migration and invasion in gastric cancer cells, and it can be significantly repressed by E26 transformation-specific sequence (ETS)-2 [30].

Although much is known about the aberrant expression of miR-196a and miR-196b in human CRC, little is known about the clinical relevance of such aberrations. In the current study, our data, based on a large cohort of patients with CRC, confirmed the upregulation of both miR-196a and miR-196b in CRC tissues compared with adjacent normal colorectal mucosa. We also found that miR-196a and miR-196b expression levels were both elevated in CRC tissues with positive lymph node metastasis, advanced TNM stage and poor histological grade, which are all parameters representing tumor aggressiveness. The findings that both miR-196a and miR-196b might be implicated in tumorigenesis and tumor progression of CRC, and that they are located at the same gene cluster, allowed us to hypothesize that the miR-196a/miR-196b signature could play an important role in CRC. We demonstrated that colorectal tissues with miR-196a-high and/or miR-196b-high expression had highly invasive and metastatic potentials compared with other expression patterns. Moreover, miR-196a-high/miR-196b-high status was specifically correlated with the shortest DFS and OS. The Cox proportional hazard model demonstrated that miR-196a-high/miR-196b-high CRC had higher relative risk of death than other expression patterns, implying that a combined analysis of miR-196a and miR-196b expression status may enhance our accuracy in identifying patients at high risk of aggressive tumor progression and poor prognosis, and hence provide useful information for clinical management.

In conclusion, our findings reveal that both miR-196a and miR-196b may be correlated with aggressive progression and unfavorable clinical outcome in CRC patients. Combined expression of miR-196a and miR-196b may be a promising biomarker in identifying a poor prognosis group of CRC.

## Materials and methods

### Study population

The protocol of the present study was approved by the Institutional Research Ethics Board of Xiangya Hospital of Central South University. A written informed consent was obtained from each subject enrolled in the present study.

One hundred and twenty-six patients with CRC (76 men and 50 women), who underwent a potentially curative surgical resection defined as removal of all macroscopic tumor masses, absence of microscopic residual tumor, histologically confirmed negative resection margins, and extension of lymphadenectomy beyond involved nodes, at the Department of General Surgery, Xiangya Hospital, Central South University, between February 2001 and January 2003 were enrolled in the present study. The median age of patients at the time of admission was 66 years, range 22–82 years. None of the patients had received either radiotherapy or chemotherapy preoperatively. All diagnoses were confirmed histopathologically, and the paraffin blocks of all primary tumor specimens were available for analysis. Only patients with sporadic CRC were selected for our analysis, and patients with a positive medical history for hereditary non-polyposis CRC or familial adenomatous polyposis were excluded at the time of patients’ collection. The sixth edition of American Joint Committee on Cancer (AJCC) TNM staging system was used for tumor staging. The detail information on the clinicopathologic characteristics of all 126 patients with CRC in this study was shown in Table 1. In addition, 126 adjacent colorectal mucosa were surgically excised from the same patients and confirmed as normal by histology.

All 126 patients with CRC were given a follow-up exam (direct evaluation or phone interview) ranging from 1 to 10 years (median 6.18 years). Patients who died from diseases other than CRC or from unexpected events were excluded from the case collection in this study. For the analysis of survival and follow-up, the date of curative surgery was used to represent the beginning of the follow-up period. DFS was defined as the length of time from curative surgery to the first tumor recurrence and distant metastasis. OS was defined as the time from curative surgery to death from any cause.

### Quantitative real time-PCR (qRT-PCR)

Expression levels of miR-196a and miR-196b in 126 pairs of fresh tumor samples matched with adjacent colorectal mucosa obtained from 126 patients with CRC were detected by qRT-PCR. Total RNA was isolated with Trizol (Invitrogen, Carlsbad, CA) according to the manufacturer’s instructions. First-strand cDNA synthesis was carried out using TaqMan MicroRNA Reverse Transcription kit and RT primers specific to the various miRNAs (Applied Biosystems, Foster City, CA, USA). Real-time PCR was carried out with TaqMan Universal Master Mix II and TaqMan MicroRNA Assay Mix on an ABI PRISM 7500 (Applied Biosystems). U6 RNA was used as an internal control to correct variations in the experiment. The RT-primer for miR-196a: 5′- GTC AGA AGG AAT GAT GCA CAG CCA ACA ACA -3′; The PCR primer for miR-196a: forward 5′- CGT CAG AAG GAA TGA TGC ACA G -3′, reverse 5′- ACC TGC GTA GGT AGT TTC ATG T -3′; The RT-primer for miR-196b: 5′- TAG GTA GTT TCC TGT TGT TGG G -3′; The PCR primer for miR-196b: forward 5′- TAG GTA CCA CTT TAT CCC GTT CAC CA -3′, reverse 5′- ATC TCG AGG CAG GGA GAG AGG AAT AA -3′; The PCR primer for U6: forward 5′-CTC GCT TCG GCA GCA CA-3′, reverse 5′-AAC GCT TCA CGA ATT TGC GT-3′. Expression of miRNA was defined based on the threshold cycle (Ct). The 2^-ΔΔCT^ method was used to calculate the relative expression levels of miR-196a or miR-196b. Each sample was assessed in triplicate for each miRNA.

### Statistical analysis

Statistical analyses were performed using the SPSS software package (version 13.0; SPSS Inc, IL, USA). miR-196a or miR-196b expression in CRC tissues and adjacent normal mucosa was compared by paired two tailed t tests. The χ^2^-test or Fisher’s exact test was employed to evaluate the association between miR-196a/miR-196b expression and clinicpathological characteristics. The correlation between miR-196a and miR-196b expression was determined by Spearman’s rank correlation analysis. The survival analysis was estimated by the Kaplan-Meier method and was compared by using the log-rank test. Multivariate analysis was performed using the Cox proportional hazard model. Differences were considered significant if *P* <0.05.
